# Perineal ultrasound to assess the urethral spatial movement in stress urinary incontinence in women

**DOI:** 10.1186/s12894-023-01220-x

**Published:** 2023-03-27

**Authors:** Binbin Dong, Yingqiu Shi, Yin Chen, Ming Liu, Xiaoming Lu, Yadong Liu

**Affiliations:** 1 Department of Urology, The Sixth Affiliated Hospital of Nantong University, Nantong, Jiangsu Province China; 2 Department of Urology, Yancheng Third People’s Hospital, Yancheng, Jiangsu Province China

**Keywords:** Urinary incontinence, Perineum, Ultrasonography, Urethra

## Abstract

**Background:**

Perineal ultrasound as a non-invasive method for the diagnosis of female stress urinary incontinence has attracted more and more attention. However, the criteria for stress urinary incontinence in women using perineal ultrasound have not been fully established. Our study aimed to evaluate characteristics of the urethral spatial movement with perineal ultrasonography.

**Methods:**

A total of 136 female patients with stress urinary incontinence and 44 controls were enrolled. Stress urinary incontinence was diagnosed using the International Consultation on Incontinence Questionnaire Short Form, medical history and physical examination, and severity was assessed using a 1 h pad test. We described the mobility of four equidistant points (A–D) located along the urethra length. The retrovesical and urethral rotation angles were measured using perineal ultrasonography at rest and during the maximal Valsalva maneuver.

**Results:**

Patients with stress urinary incontinence showed a more significant vertical movement at Points A, B and C than controls. The mean variations in the retrovesical angle were significantly larger in patients with stress urinary incontinence at rest and during the Valsalva maneuver than in controls (21.0 ± 16.5° vs. 14.7 ± 20.1°, respectively). The cut-off value for the retrovesical angle variation was 10.7° with 72% sensitivity and 54% specificity. There was a receiver-operating characteristic curve area of 0.73 and 0.72 for Points A and B, respectively. A cut-off of 10.8 mm, and 9.4 mm provided 71% sensitivity and 68% specificity and 67% sensitivity and 75% specificity, respectively.

**Conclusions:**

The spatial movement of the bladder neck and proximal urethra, and variations in the retrovesical angle may be correlated with clinical symptoms and facilitate to the assessment of SUI.

## Introduction

The International Continmence Society (ICS) defines urinary incontinence (UI) as “any involuntary leakage of urine” or “urine leakage seen during examination”, that bladder pressure exceeds the pressure of the urethra has the capacity to closed [1]. Nearly half of adult women experience UI [2]. One study reported that the incidence of UI in women increases from 20.5% at age 15 to 68.8% at menopause [3]. The main types of UI include stress (SUI) and urge (UUI) urinary incontinence. SUI is the most prevalent type, especially in younger women [4, 5]. Approximately 6.8 million women had a primary diagnosis or chief complaint of UI in the United States in 2009/2010 [6]. Only 25% of incontinent women seek help and less than half receive treatment. [7]

Conventional diagnostic methods, including medical history and physical examination, UI questionnaires, urinalysis, and Q-tip testing, have certain limitations. Therefore, highly sensitive and specific tools are crucial for the correct diagnosis of UI. Loss of urethra function and morphological changes are important in the occurrence of urinary incontinence. Perineal ultrasonography (US) is a non-invasive test that depicts anatomical structures of the bladder and urethra and identifies urine leakage [8]. However, in previous studies, the indexes of urethral mobility may vary due to the difference of measurement methods and conditions [9–10], while every method has its emphasis.

In this study, perineal US were applied to focus on evaluate the two dimensions movement feature of the urethra between SUI and NSUI, meanwhile to assess the predictive value by the measurement method in female patients with SUI.

## Methods

We registered 144 female patients with SUI and 50 controls between October 2020 and August 2021 in Chinese population. All patients provided informed consent and this analysis was approved by Yancheng third people’s hospital Human Research Ethics Committee. Assessments included medical history, physical examination, 3-day bladder diary, International Consultation on Incontinence Questionnaire Short Form (ICIQ-SF), urine routine, and biochemical examination. Patients with neuropsychiatric disorders, cognitive disorders, diabetes mellitus, urogenital surgery, pelvic irradiation, genitourinary tumors, pelvic organ prolapse, and urinary tract infections were excluded. The control group was women without pelvic disease and urinary incontinence during the same period. Finally, 136 female patients with SUI (SUI group) and 44 controls (NSUI group) were enrolled. Both groups underwent perineal US, and the SUI group additionally underwent 1 h pad test.

### Perineal US

**Fig. 1:**
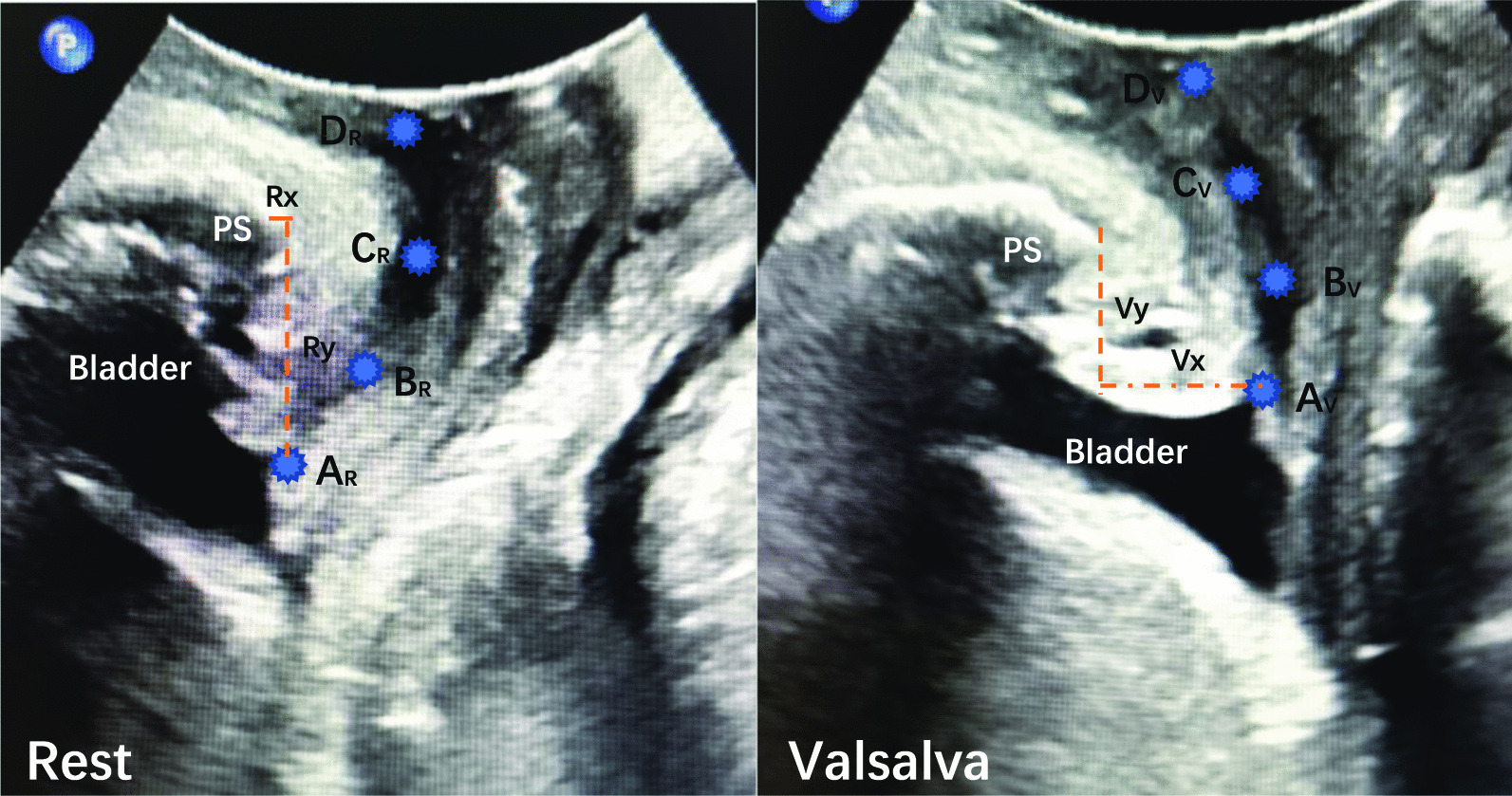
The urethra image was measured by perineal ultrasound image in the mid-sagittal plane at rest (left) and on Valsalva (right). The urethra is divided into three segments, defined by four points from the bladder neck (point A) to the external meatus (point D). The inferoposterior margin of the symphysis pubis serves as reference. The motion of Point A is shown as an example. A_R_ was the position of point A at rest, and A_V_ was the position of point A on maximal Valsalva manoeuver

**Fig. 2:**
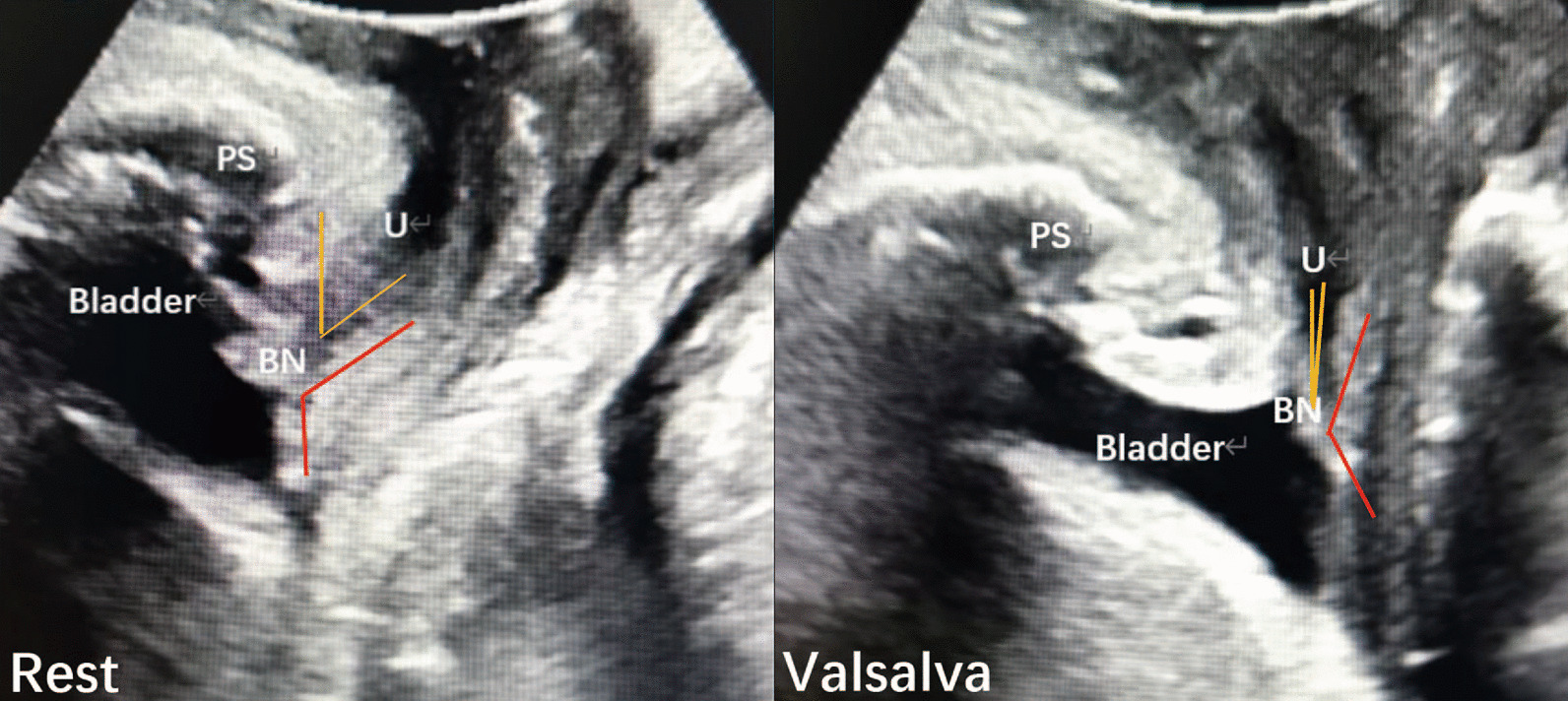
Perineal ultrasoubd image of the pelvic organs. Anatomical landmarks are the pubic symphysis (PS) and bladder with bladder neck (BN). The urethra (U). The posterior urethrovesical angle (the red arrow) and urethral inclination angle (the yellow arrow) was measured both at rest and on maximal Valsalva manoeuver

Perineal US was performed using the Voluson E8 system (GE Healthcare, Milwaukee, WI, USA) equipped with a 1–5 MHz curved array transducer placed on the perineum in a sagittal direction. And Residual urine volume should not exceed 100 ml. Using a coordinate system based on the origin of the lower margin of the symphysis pubis, manual measurements were obtained using the GE Kretz 4D View version 5.0 (GE Kretz, Zipf, Austria). US data were obtained at rest and during at least three Valsalva maneuvers held for 5–6 s, with the most effective measurement used for analysis. To determine the urethral motion profile, the urethral length was manually traced on US images in the mid-sagittal plane, both at rest and during the maximal Valsalva maneuver. This traced length was then divided into three segments with four equidistant points (A, B, C, D), from Point A (bladder neck) to Point D (external urethral meatus), as shown in Fig. 1. Mobility vector distances (MVD) for these points (AMVD, BMVD, CMVD, DMVD) were determined using the formula, $\sqrt{{(Vx-Rx)}^{2}+({Vy-Ry)}^{2}}$ which allowed us to describe the difference between point coordinates from the lower margin of the symphysis pubis (x = horizontal distance; y = vertical distance) at rest (R) and during the Valsalva maneuver (V). The retrovesical angle (RVA; the angle formed by the urethral axis and a line drawn tangentially to the posterior edge of the bladder base near the bladder neck) and the urethral inclination angle (the angle between the urethral axis and the vertical axis) were measured both at rest and during the Valsalva maneuver. The urethral rotation angle (URA), which is the difference in urethral inclination angles at rest and during the Valsalva maneuver, was also measured (Figs. 1, 2). Image analysis: The images were analyzed by 2 physicians with at least 2 years of experience in real-time ultrasound and pelvic floor ultrasound, and the results were determined by consultation of the 2 physicians.

### Statistical analyses

Statistical evaluation was performed using SPSS 21 for Windows (SPSS, Chicago, IL, USA). Data are presented as mean ± standard deviation. The results of perineal ultrasonography in our study were compared using the paired t-test for normally distributed data and the Wilcoxon test for non-normally distributed data. Two-tailed *P* < 0.05 was considered statistically significant. We constructed receiver-operator characteristic (ROC) curves to choose cut-off limits of urethral mobility to discriminate between normal and incontinent women.

## Results

The mean age in the SUI and NSUI groups was 54.4 ± 9.5 (range, 29–78) and 49.3 ± 10.4 (range, 29–70) years, respectively. Seventy-five percent of women with SUI were vaginally parous [mean parity 2.1 ± 1.4 (range: 1–7)], while 68% of NSUI women were vaginally parous [2.0 ± 1.5 (0–5)]. Body mass index (BMI) was 22.5 ± 3.4 (range: 17.3–29.7 kg/m2) in the SUI group and 22.4 ± 3.1 (17.5–30.2) in the NSUI group (Table 1).

**Table 1:** Main characteristics of our patients and comparison of general information between groups SUI and NSUI

Group	BMI (kg/m2)	Age (years)	Parity
SUI	22.5 ± 3.4	54.4 ± 9.5	2.1 ± 1.4
NSUI	22.4 ± 3.1	49.3 ± 10.4	2.0 ± 1.5
*P*	> 0.05	> 0.05	> 0.05

*BMI* body mass index, comparison of BMI and parity between groups SUI and NSUI

Based on the 1-h pad test, 73 patients had moderate to severe SUI (> 10 g) and 63 patients had mild SUI (< 10 g). In the moderate to severe SUI group, the mean age was 54 ± 9.4 (33–75) years, mean BMI was 22.7 ± 3.6 (17.8–28.8 kg/m2), mean vaginal parity was 2.1 ± 1.4 (1–7), and mean ICIQ-SF score was 11.9 ± 4.8 (0–21). In the mild SUI group, the mean age was 55 ± 9.7 (29–78) years, mean BMI was 22.6 ± 3.2 (17.3–29.7 kg/m^2^), mean vaginal parity was 2.1 ± 1.4 (1–7), and mean ICIQ-SF score was 5.9 ± 6.0 (0–17). On the 1 h standardized pad test, women from the moderate to severe group leaked more urine than those from the mild group [30.3 ± 24.3 (10.8–82 g) vs. 3.0 ± 2.4 (0.27–8.7 g); *P* < 0.05] (Table 2).

**Table 2:** Main characteristics of our patients and comparison of general information between groups Moderate-severe SUI and Mild SUI

Group	BMI (kg/m2)	Age (years)	Parity	ICIQ-SF
Moderate -severe SUI group	22.7 ± 3.6	54 ± 9.4	2.1 ± 1.4	11.9 ± 4.8
Mild SUI group	22.6 ± 3.2	55 ± 9.7	2.1 ± 1.4	5.9 ± 6.0
P	> 0.05	> 0.05	> 0.05	< 0.05

*BMI* body mass index, comparison of BMI and parity between groups SUI and NSUI

The mean URA was 25.3 ± 18.4° (1–69.0°) in the SUI group and 26.3 ± 18.5° (1–67.8°) in the NSUI group (*P* > 0.05). The mean variation in RVA (RVAδ) was significantly higher in the SUI group [21.0 ± 16.5° (−28.5 to 63.5°) vs. 14.7 ± 20.1° (−30.9 to 60.9°); *P* < 0.05] (Table 3).

**Table 3:** Comparison of the angle parameters by Perineal ultrasonography between SUI and NSUI groups

Group	RVA			URA
	Rest	Valsalva	RVA_δ_	
SUI	122.5° ± 14.8°	143.4° ± 15.7°	21.0° ± 16.5°	25.3° ± 18.4°
NSUI	118.8° ± 16.1°	133.5° ± 16.1°	14.7° ± 20.1°	26.3° ± 18.5°
*P*	> 0.05	< 0.05	< 0.05	> 0.05

Urethral inclination angle was measured both at rest and on maximal Valsalva manoeuver. The RVA_δ_, which is the variation of RVA at rest and most effectively Valsalva. The urethral rotation angle (URA), which is the difference of the angles of urethral inclination angle at rest and the Valsalva manoeuver

Perineal US measurements of the mean mobility of the segmental urethra from the bladder neck (A) to the external urethral orifice (D) are shown in Table 5. The mean mobility of the bladder neck (Point A) was 14.6 ± 7.0 (2.0–33.4) mm in the SUI group and 10.0 ± 4.1 (2.0–22.3) mm in the NSUI group. The same measurements at Points B–D were 11.9 ± 5.6 (3.9–27.5), 8.5 ± 4.9 (0.4–22.5), and 6.8 ± 3.7 (1.3–21.0) mm, respectively, in the SUI group and 8.2 ± 3.4 (2.9–20.0), 7.1 ± 2.7 (0.3–13.9), and 6.1 ± 3.5 (2.2–23.0) mm, respectively, in the NSUI group. There was a generalized change of segmental urethral mobility in the SUI group; however, this only reached significance (vs. NSUI) at Points A, B and C. (Table 4).

**Table 4:** Comparison of the urethra parameters by Perineal ultrasonography between SUI and NSUI groups

Group	A_MVD_	B_MVD_	C_MVD_	D_MVD_
SUI	14.6 ± 7.0 mm	11.9 ± 5.6 mm	8.5 ± 4.9 mm	6.8 ± 3.7 mm
NSUI	10.0 ± 4.1 mm	8.2 ± 3.4 mm	7.1 ± 2.7 mm	6.1 ± 3.5 mm
*P*	< 0.05	< 0.05	< 0.05	> 0.05

A_MVD_, B_MVD_, C_MVD_, D_MVD_ are described as the difference in the distance at urethral coordinates (x coordinates, y coordinates) form the rest to Valsalva

Analysis of the characteristics of Points A, B and C in the two groups showed significant differences in the mean mobility vectors at Point A in the horizontal and vertical direction [9.5 ± 6.3 (SUI) and 6.8 ± 3.9 mm (NSUI); 9.8 ± 1.2 (SUI) and 6.1 ± 3.4 mm (NSUI), respectively, both P < 0.05] and at Point B in the horizontal and vertical direction [6.8 ± 4.0 (SUI) and 5.3 ± 2.9 (NSUI), P > 0.05; 8.9 ± 5.4 (SUI) and 5.3 ± 3.3 (NSUI), P < 0.05] and at Point C in the horizontal and vertical direction [3.8 ± 3.0 (SUI) and 4.0 ± 2.6 (NSUI), P > 0.05; 7.3 ± 4.9 (SUI) and 5.1 ± 3.2 (NSUI), P < 0.05] (Table 5).

**Table 5:** Comparison of the urethra parameters by Perineal ultrasonography between SUI and NSUI groups

Group	A		B		C	
	X_A_	Y_A_	X_B_	Y_B_	X_C_	Y_C_
SUI	9.5 ± 6.3 mm	9.8 ± 1.2 mm	6.8 ± 4.0 mm	8.9 ± 5.4 mm	3.8 ± 3.0 mm	7.3 ± 4.9 mm
NSUI	6.8 ± 3.9 mm	6.1 ± 3.4 mm	5.3 ± 2.9 mm	5.3 ± 3.3 mm	4.0 ± 2.6 mm	5.1 ± 3.2 mm
*P*	< 0.05	< 0.05	> 0.05	< 0.05	> 0.05	< 0.05

X_A_, X_B_, X_C_ are described as the absolute value of the difference in the distance at x coordinates during the rest (R_X_) and Valsalva (V_x_). Y_A_, Y_B_, Y_C_ are described as the absolute value of the difference in the distance at y coordinates during the rest (R_y_) and Valsalva (V_y_)

**Fig. 3:**
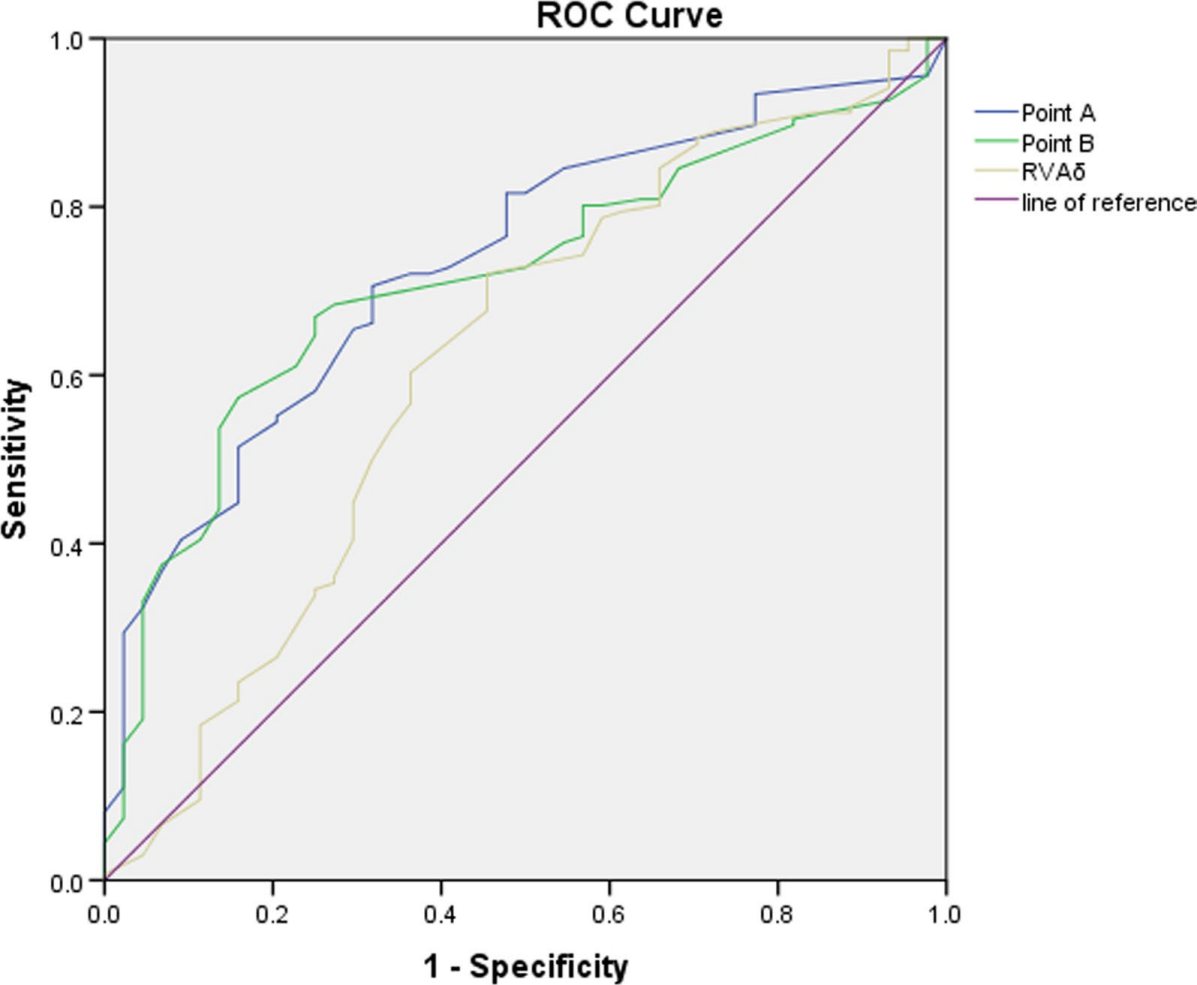
ROC curves (receiver operating curve) analyses of three parameters for predicting stress urinary incontinence by perineal ultrasonography. Point A: the mobility vector distances of A (A_MVD_); Point B: the mobility vector distances of B (B_MVD_); RVA_δ_: the mean variation of retrovesical angle on rest and most effectively Valsalva

ROC curves for RVA and segmental urethral mobility (Points A and B) were constructed to determine the cut-off values for SUI and NSUI discrimination. In the RVAδ examination, we obtained an ROC area of 0.62 [95% confidence interval (CI) 0.52–0.72] and chose 10.7° as the cut-off point with 72% sensitivity and 54% specificity. Segmental urethral mobility examination resulted in an ROC area of 0.73 (95% CI 0.66–0.81) for Point A, and a cut-off of 10.8 mm provided 71% sensitivity and 68%, specificity. We obtained an ROC area of 0.72 (95% CI 0.76–0.94) for Point B and chose 9.4 mm as the cut-off point with 67% sensitivity and 75% specificity (Fig. 3).

## Discussion

In this study, we explored the meaning of segmental urethral mobility of the urethra by perineal US. We found that the mobility in all urethral segments, especially the proximal urethra, along with the RVAδ played a significant role in the pathogenesis of SUI.

Sendag [13] reported that a descending distance of the bladder neck > 15 mm and a posterior RVA > 120° were related to poor support of the bladder neck in patients with SUI with 96% and 53% sensitivity and 85% and 100% specificity, respectively. During straining condition, SUI women demonstrated > 1 cm of caudal motion of their urethrovesical junction by transrectal US, while control had < 1 cm of that [14]. Another study speculated that a bladder neck distance (BND) ≥ 25 mm may be abnormal and associated with SUI [15]. Our results showed that the sensitivity and specificity of cut-off point A (bladder neck > 10.8 mm) as a diagnostic criterion were 71% and 68%, respectively. The inter-study differences in the results related to the descending distance of the bladder neck may be due to different measurement methods, as well as race. We combined the vertical and horizontal motion of the bladder neck, which may provide a better representation of the spatial movement in the bladder neck. However, a recent study confirmed that BND alone cannot comprehensively evaluate SUI [16]. Similar to our results, one Chinese report suggested that the specificity of BND in SUI diagnosis was only 68.9% [17]. Thus, we need to explore the abnormal urethral support structure to understand the etiology and pathogenesis of SUI. Most studies have focused on bladder neck mobility and provided little information about the rest of the urethra [18]

The phenomenon where there is a rotational movement around the tip of the symphysis pubis during stress was observed on US in all patients. This rotational movement occurs in a cranial-to-caudal and dorsal-to-ventral direction, which is why we decided to use a mathematical description of urethral mobility. Our results showed that this change in mobility was similar for all urethral segments and was not restricted to the mid-urethra, suggesting that mobility at all stages of the urethra should be considered in UI studies. One previous study divided the urethra into five equal segments with six points from the bladder neck to the external urethral meatus, and results showed that the proximal urethra was consistently more mobile than the distal urethra (*P* < 0.001) [19]. However, they did not compare horizontal movement. In our study, the mean mobility and vertical distance of Points A (Y_A_), B (Y_B_) and C(Yc) in SUI were much greater than those in NSUI, and horizontal movement was one of the most influential factors. The horizontal distance of Point A in SUI was significantly greater than that in NSUI. However, there was no significant difference between SUI and NSUI at Point B and C. We speculated that there is more horizontal and vertical movement of the urethra closer to the neck of the bladder. Points D were the least mobile, suggesting that loss of proximal urethral stability leads to abnormalities in urethral mobility, resulting in SUI.

SUI is related to both urethral support and morphological changes in the junction of the urethra and bladder. In this study, there was no obvious change in urethral inclination, but the mean RVAδ at rest and during the Valsalva maneuver was clearly different in SUI and NSUI patients. A similar study showed that SUI was significantly associated with RVAδ. The mean RVA during maximum Valsalva was 152°, which was similar to our results (most effective Valsalva maneuver 143.4 ± 15.7°). We believe that the difference in RVA may be related to bladder volume and Valsalva maneuvers during detection. We further tested the mean RVAδ at rest and during the Valsalva maneuver, and speculated that this indicator may better explain the differences in bladder volumes. However, a previous study confirmed that there are no differences in BND, URA, or posterior urethro-vesical angle (PUVA) for different bladder capacities (100–500 ml) [20]. One study [21] revealed that BND, URA, and RVA were not significantly different in mild to moderate and severe patients. Thus, perineal US is a useful tool for identifying SUI, but not its severity. Real time assessment of bladder neck and proximal urethra behavior using transperineal US indicated that the median RVA (166°) in the standing position was significantly greater than RVA (133°) in the supine position, and the median URA of 35° was significantly smaller than that of 64° in the supine position [22]. In our study, the measurements were performed with patients in the supine position, and the mean URA of 25.3 ± 18.4° and mean RVA of 143.4 ± 15.7° were smaller than reported by the previously mentioned study. This may be due to different patient variables and the standard of Valsalva maneuvers. We found no obvious difference in RVA at rest between SUI and NSUI, but there was a significant difference in RVA during the Valsalva maneuver. Another report showed similar results whereby the difference in PUVA at rest and during Valsalva maneuver in SUI was significantly higher than in NSUI [23].

In terms of BND results (optimal cut-off, 24 mm), the area under the ROC curve (AUC) value was 0.804, with 66.4% sensitivity and 84.5% specificity^24^. In our results, the ROC area of Point A was 0.73 with the optimal cut-off 10.8 mm, and this cut-off point provided 71% sensitivity and 68%, specificity. Moreover, Point B (cut-off 9.4 mm) provided 67% sensitivity and 75% specificity. We believe that bladder neck activity and the first one-third of the length from the urethra to the bladder neck has value in predicting UI. The AUC value of RVAδ was 0.62 (cut-off 10.7°), with 72% sensitivity and 54% specificity. This represents a weak predictive index for SUI.

## Conclusions

In our study, the horizontal and vertical direction movement of proximal urethra and RVAδ were the variables most closely associated with SUI. These parameters may be correlated with clinical symptoms and facilitate to the assessment of SUI.

## Data Availability

The datasets used and/or analysed during the current study available from the corresponding author on reasonable request. The images in study were appropriated permission by patients.

## Abbreviations

AUC: Area under the ROC curve
BMI: Body mass index
BND: Bladder neck distance
ICIQ-SF: International Incontinence Questionnaire Short Form
ICS: International Continence Society
MVD: Mobility vector distances
PUVA: Posterior urethro-vesical angle
ROC: Receiver-operator characteristic
RVA: Retrovesical angle
RVAδ: Mean variation in RVA
UDS: Urodynamic study
UI: Urinary incontinence
URA: Urethral rotation angle
US: Ultrasonography
